# Sensor-Based Gym Physical Exercise Recognition: Data Acquisition and Experiments

**DOI:** 10.3390/s22072489

**Published:** 2022-03-24

**Authors:** Afzaal Hussain, Kashif Zafar, Abdul Rauf Baig, Riyad Almakki, Lulwah AlSuwaidan, Shakir Khan

**Affiliations:** 1 Department of Computer Science, National University of Computer and Emerging Sciences, Islamabad 44000, Pakistan; l155360@lhr.nu.edu.pk or kashif.zafar@nu.edu.pk (K.Z.); 2 Department of Information Technology, Government College University, Faisalabad 38000, Pakistan; 3 Department of Information Systems, College of Computer and Information Sciences, Imam Mohammad Ibn Saud Islamic University (IMSIU), Riyadh 11432, Saudi Arabia; ralmakki@imamu.edu.sa; 4 Department of Information Management, College of Computer and Information Sciences, Imam Mohammad Ibn Saud Islamic University (IMSIU), Riyadh 11432, Saudi Arabia; lnsuwaidan@imamu.edu.sa (L.A.); sgkhan@imamu.edu.sa (S.K.)

**Keywords:** Internet of Things (IoT), smart sensor, inertial sensor, gym exercise recognition, human activity recognition, LSTM

## Abstract

Automatic tracking and quantification of exercises not only helps in motivating people but also contributes towards improving health conditions. Weight training, in addition to aerobic exercises, is an important component of a balanced exercise program. Excellent trackers are available for aerobic exercises but, in contrast, tracking free weight exercises is still performed manually. This study presents the details of our data acquisition effort using a single chest-mounted tri-axial accelerometer, followed by a novel method for the recognition of a wide range of gym-based free weight exercises. Exercises are recognized using LSTM neural networks and the reported results confirm the feasibility of the proposed approach. We train and test several LSTM-based gym exercise recognition models. More specifically, in one set of experiments, we experiment with separate models, one for each muscle group. In another experiment, we develop a universal model for all exercises. We believe that the promising results will potentially contribute to the vision of an automated system for comprehensive monitoring and analysis of gym-based exercises and create a new experience for exercising by freeing the exerciser from manual record-keeping.

## 1. Introduction

Human activity recognition has generated a lot of interest in recent years [1,2,3]. Gym exercise recognition comes within the scope of this topic and has two major applications: automatic logging of exercises, relieving the trainer from manual entry, and real-time availability of an honest record to the trainer’s coaches and doctors. Even though these two applications warrant exclusive and dedicated research, surprisingly very little work has been carried out on this topic.

To begin with, let us note that a human being is capable of activities at several different levels; e.g., mental, emotional, and physical. In the present research, we limit ourselves to physical activities, and more specifically to physical exercises. A physical exercise can be any activity that enhances or maintains a practitioner’s health and fitness. One way to categorize the exercises is by defining them as flexibility exercises, aerobic exercises, and anaerobic exercises. We are focusing on anaerobic exercises performed by weight trainers in a typical gym setting. A gym, or gymnasium in long form, is an indoor sports facility. A gym may have a wide range of facilities, but we are limiting ourselves to equipment and machines used by weight trainers. We use the term weight trainers in a broad sense and mean to include body builders, strength trainers, weight lifters, and practitioners of any other sport who use free weights and weight machines in their training routine.

The basic modalities used for human activity recognition are vision and non-vision (i.e., sensor) and their hybrid combination [4]. Traditionally, the vision-based approach relies on video cameras and depth cameras. The recent advances in the wearable camera technology have the potential to take this approach to new levels. The sensor-based approach can be categorized on the basis of sensor location into wearable, ambient, and their hybrid combinations. All the approaches have their pros and cons. We limit ourselves to wearable sensors.

The current research presents an architecture for gym-exercise-specific data acquisition and recognition of exercises. A single smart sensor is used to collect data. The raw time series data are fed to the long short-term memory (LSTM) neural network based on sliding window approach to recognize exercises. The exercise-specific data collection and analysis is helpful in analyzing the impact of each exercise on athletes body. This can later serve as a system for athletes’ health state analysis, comfort analysis, or it can serve as a recommendation system for upcoming athletes.

The rest of the paper is organized as follows. We present the literature survey of the gym exercise recognition research in Section 2. The details of our collected dataset are given in Section 3. Section 4 presents our experimental design choices selected from the different possibilities that exist and notes some of the results that we have obtained. Section 5 lists some possible uses of our dataset. A discussion of the study is presented in Section 6. We conclude and suggest future work in Section 7.

## 2. Related Work

As stated above, our purpose is to attempt gym exercise recognition based on data collected from wearable sensors. To place our work into context, the following research related to gym activity recognition is worth mentioning.

In [5], mechanisms for tracking free weight exercises are studied. They aim to automatically recognize what type of exercise is being performed and how many repetitions have been performed. The collected dataset comprises a total of nine common free weight dumbbell exercises, representative for each muscle group, performed in a gym environment. The posture while performing these exercises can be standing, sitting, or lying down. A three-axis accelerometer was incorporated into a workout glove to track hand movements and another accelerometer was attached to the user’s waist to track body posture.

The research reported in [6] exploited built-in accelerometers of smartphones to capture exercise data, and they aimed to detect the start and end times of exercise repetitions from a continuous stream of acceleration data and consequently were able to track the number and duration of repetitions. They conducted experiments in two distinct scenarios, constrained and unconstrained environment. The equipment used for the exercises were resistance bands, free weights, and body weight. The constrained environment was a gym, and exercises were performed using free weight machines. Unconstrained exercises were performed without any weight machines and in different places, such as homes and parks. Two series, each having ten repetitions of several exercises, were performed in both types of environments. The smartphone was placed on the top of weights in the constrained environment scenarios. The placement of the smartphone in the unconstrained environments was either on the subject’s wrist or ankle, depending upon the exercise.

Feedback and guidance after assessment of the quality of free weight exercises is the main target of [7]. In this research, after defining what is meant by quality of exercise execution, they investigated three aspects pertaining to qualitative activity recognition, specification of correct execution, detection of execution mistakes, and provision of feedback to the exerciser. The dataset comprised data from only one weight lifting exercise: the unilateral dumbbell bicep curl. The data were collected with the help of six subjects performing five different variations of this exercise with 10 repetitions in each variation. One of them was the correct execution of the exercise, while the other four variations were prevalent mistaken ways. Four inertial measurement units (IMU) were used and they were placed in the subject’s glove, armband, lumbar belt, and dumbbell. These sensors provided not only tri-axial acceleration data, but also gyroscope and magnetometer data. The dataset is publicly available in the UCI Machine Learning repository [8].

Inertial measurement units (IMUs) are low-power devices with sensors consisting of accelerometers and gyroscopes. The work reported in [9] aimed at exploring the problem of automated tracking and analyzing of weight training exercises using IMUs. An accurate and fast tracking of selected weight training exercises was shown to be possible by targeting seven dumbbell exercises. These exercises cover the most important upper body muscle sections. Users performed these exercises using a dumbbell equipped with the hardware prototype. Automatic tracking and quantification of the exercises had been attempted in [10]. They analyzed data coming from wearable IMUs and attempted classification and counting of exercises. A circuit of nine exercises were targeted with a group of seven volunteers. The IMU sensor used was worn on the wrist by the subjects.

A free weight exercise monitoring system (FEMO) [11] provided an integrated free weight exercise monitoring, quality analysis, and feedback system. They focused on ten common and representative free weight activities that can train different parts of the muscle groups. The data collection was carried out with the help of fifteen volunteers. The sensors used in the system are passive RFID tags attached on the dumbbells. An implementation of a micro-watt-level power consumption is presented in [12], seven popular gym workouts were recognized and counting was achieved based on a body-capacitance-based sensor with three different body positions. An RFID and computer-vision-technique-based deeper gym exercise monitoring system (DEEM) was introduced in [13]. The DEEM system can determine the user and the objects that users hold. An accelerometer-based gym exercise recognition system named Fine-Fit was proposed in [14], which is useful in monitoring gym exercises and avoiding muscle injury by assessing non-standard actions. The Fine-Fit system can sense the body movement and muscle vibration simultaneously.

The above described research is mostly related to weight exercises performed in a gym environment. The experiments and reported results led us to believe that there is much room for a system that can cater for a broad range of free weight exercises. Such a system needs to have reasonable accuracy and should be tested on a large dataset consisting of a wide range of gym-based exercises. In the present research, we report our efforts towards the development of such a system.

On a broader scale, a body of literature is available on detection and analysis of sports-related activities and fitness exercises. Interested readers may refer to any of the numerous surveys that are available. An example of such surveys is [15,16]. Furthermore, there are numerous excellent works in the parent field of activity recognition. A few of them are [1,2,3,17,18,19,20,21,22,23,24,25].

## 3. Dataset Acquisition

In this paper we focus on gym exercise recognition, and our overall objective is to facilitate weight trainers as much as possible by automation implemented with the recent advances in Internet of Things (IoT) and related technologies. A vast majority of human activity research deals with only a handful of daily exercises [4,8,26]. These exercises are very different from the exercises performed in a gym environment with gym equipment. Thus, we collected our own dataset by targeting six muscle group and forty-two exercises. In this paper, we extend our earlier work reported in [27]. The presented approach will contribute towards the development of apps that automatically track free weight exercises. The usage of such apps can be very convenient in the fields of sports and health.

The data collection process for human activity recognition is a challenging task, particularly when it involves gym-based free weight exercises. It is difficult to find appropriate participants that are practitioners of the sport, prepare them for data collection, and keep them motivated for the entirety of the process. Furthermore, the high cost of sensors, adherence to hygiene when using the same sensor on multiple users, and privacy issues complicates the task. In this section, we describe all aspects of our data acquisition and transformation process.

### 3.1. The Gym Exercises

This study includes forty-two physical exercises commonly performed by practitioners of body building and muscle training programs using free weights [28]. The approval of the data collection process was obtained from the Government College University, Faisalabad, Pakistan’s Ethical Review Committee, and all the subjects submitted their written consent and willingness to participate in the experiment. The descriptions of these exercises are widely known [29,30]. The explanations of some of the terms used by weight trainers are as follows. The fundamental unit of an exercise is called a rep (short for repetition). It is one complete motion of an exercise, analogous to taking a step while walking or running. Beginning from a starting position, the practitioner follows a sequence of motions and returns to the starting position. A group of consecutive repetitions of an exercise is called a set. There is a short period of rest between two sets. Typically, weight trainers take 30 s to 3 min of rest between two consecutive sets. This interval of rest is essential because it allows the targeted muscle to recover from fatigue of the previous set and prepare for the next set. Weight trainers typically complete all the sets of an exercise in a sequential manner and then start the next exercise. A workout is the complete group of exercises performed during a session, and most of the practitioners have one session in a day.

**Figure 1:**
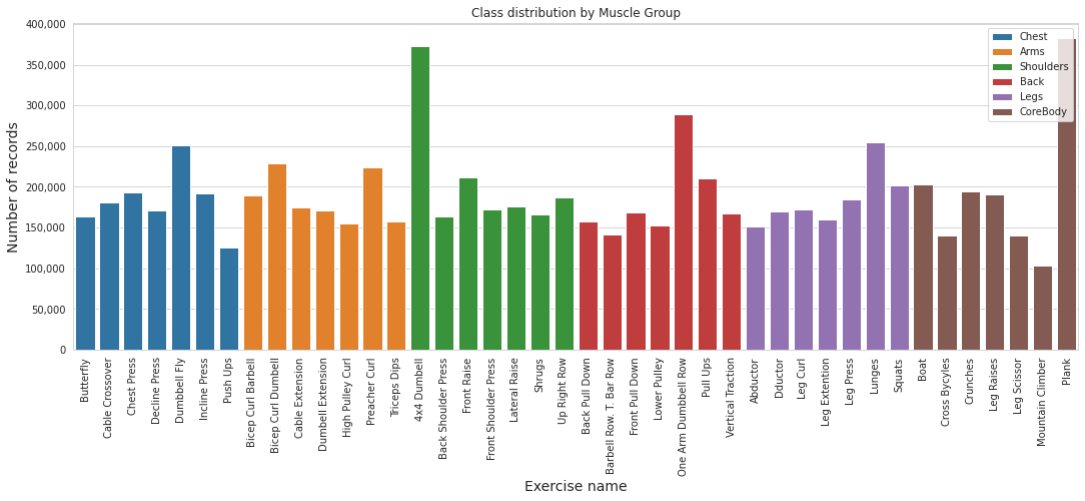
Exercise-wise instance count.

The 42 exercises covered in this study are listed in Table 1, and their instance count is depicted in Figure 1. The *x*-axis in Figure 1 lists the names of exercises based on muscle group, and exercise-wise instance count is presented on the *y*-axis. All of these exercises are well known and a selection of them are included in a typical workout. Some of these exercises are basic and are performed by all weight trainers, from beginner to advanced level. Others are usually included in the workouts of intermediate- and advanced-level practitioners of the sport.

The weight trainers typically target one or two muscle groups on any given day. We placed the exercises for each muscle group into a separate workout. These groups are chest, arms, shoulder, back, and legs. One last group is added that have exercises that have a good effect on several muscle groups. For our data collection, one workout was performed in one session. There are six workouts for six consecutive days of the week, with the seventh day as the rest day. Most of these exercises need weights mounted on barbells or dumbbells, while some of them need extra equipment. Furthermore, some exercises are performed standing, while others are performed sitting, and a few while lying down on a bench.

**Figure 2:**
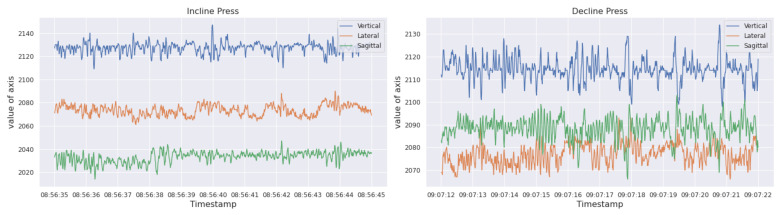
Visual inspection of acceleration data for incline press and decline press activity from chest workout.

Previously, there have been several successful attempts for assorted human activity recognition tasks by using a single, chest-mounted acceleration sensor [31,32]. It led us to speculate that gym-based free weight exercise recognition can also be attempted by such an approach. To lend credence to our speculation, we visually inspected the acceleration data for each exercise and were able to differentiate several exercises from one another. As an example, data for two different exercises from the same group are shown in Figure 2, where the *x*-axis represents the time of reception of signal and the y-axis represents the value. Each vertical, lateral, and sagittal axis of the signal has discriminating characteristics for the two exercises. A commercially available device, Zephyr BioHarness 3 (BH3), was used for the data collection process. The BH3 device has many basic and advanced features based on several sensors [33].

### 3.2. The Data Collection Process

As discussed in the previous section, this study involves forty-two exercises arranged into six muscle groups, and each muscle group contains seven exercises. A day is associated with all the exercises belonging to a muscle group; thus we have six muscle groups, and all the seven exercises of a muscle group are performed in a day. The complete set of forty exercises are performed in a week with one day as rest. We collected two types of data: body movement data and exercise-related data. The body movement data was collected using the Zephyr BioHarness (BH3) device. The BH3 device was strapped on the chest of the subjects during each exercise. The position of the BioHarness was approximately at the pectoral muscle, for all data collection. Our experiments show that the device is not sensitive to sensor positioning (up to a reasonable extent). Small differences in the position of the device do not disrupt recognition results [34,35].

Choice of a good sampling rate is very important. Too high a rate is wasteful and too low a rate will not be able to capture the necessary information. It has been previously shown that a 20 Hz sampling rate is ample for general purpose activity recognition by accelerometers [36]. We use a higher rate of 100 Hz because gym exercises are usually more rapid. The rate may be higher than necessary, but data at a higher rate can be downsampled later on to find an optimal sampling rate.

The acquired data are from four male subjects, aged between 18 and 25. The subjects performed the seven exercises in the workout planned for that day. Each exercise was performed three times (i.e., three consecutive sets for each exercise) in a day. There was a small resting period between two sets of an exercise. Since the strength and endurance for each subject was different from one another, the weights used by them varied even for the same exercise. For example, a subject might have used 50 kg weight for an exercise and another one 30 kg for the same exercise, but both of them retained the same weight for all the three sets and performed 10 reps per set. Since the exercises are different and the capacity of the subjects is also different, the samples are not temporally homogeneous. The same exercise by different subjects, and even by the same subject, takes different times to complete. Similarly, two different exercises may also take different times due to their nature of execution being different.

A manual record was made to note the subject’s name, exercise name, start and end times, repetitions, weights, number of sets, and any exercise equipment that was used. A mobile app was used for this data entry. This data is useful for segmenting the raw data into proper samples and for annotating it properly. Relevant basic and health-related information regarding the participants was noted at the beginning of the entire data acquisition process. This included participants’ gender, age, height, weight, any known health issues, etc.

The workout plan is for six weeks. Every week, six days are for exercises and one day is for rest. On each of the exercise days, a workout on the seven exercises for a muscle group was scheduled, with three repetitions of each exercise. Since we have four subjects and each one performed three sets of seven exercises each day, we have a total data of 3024 sets for the 6 weeks. The average time spent by each subject in the gym for data acquisition was around 70 min on a daily basis. We have a gap of six days between the same exercise because this is how practitioners of this sport perform their workouts, though it is not necessary for exercise recognition.

At the beginning of the program we had 21 subjects. Ten subjects left within the first few days and we had 11 subjects at the end of first week. Out of these 11, only four completed the entire 6-week program. The main factor for the dropout was the commitment required on a daily basis for six weeks.

BH3 has internal storage for data and it proved to be sufficient for a single day of data from all the subjects. At the end of the day, the collected data were transferred via a USB connection to a laptop for more permanent storage.

The data collection was conducted primarily in an average gym equipped with only the basic equipment, under the supervision of a researcher. For some exercises, the subjects had to venture outside the gym, accompanied by the researcher.

### 3.3. The Unprocessed Data

The data are generated in their raw form when exercises are performed by our subjects. Three real numbers are provided by the tri-axial accelerometer for each of the vertical, lateral, and sagittal axes. As mentioned before, each subject performs seven different exercises (one workout) on a given day. Each exercise is composed of three consecutive sets, with a small rest interval between the sets. A raw data file is created for each workout of each subject. We have a total of 144 raw, unprocessed, data files (4 subjects × 6 workouts × 6 weeks) and each file contains 21 complete data samples (7 exercises × 3 sets). The format of the data file is <timestamp, vertical, lateral, sagittal>, as depicted in Table 2, and the sampling rate for data collection is 100 Hz.

The weights used vary with each subject and exercise and are recorded manually using mobile application. The start and finish time of each set is also recorded. Furthermore, for completeness of data, we record the number of sets and the reps even though they are constant for the entire data acquisition. These mobile-application-based recorded data are concatenated with the automatically acquired data by using the subject-ID and the time stamp information. Table 3 depicts how the labeled accelerometer and mobile application dataset looks, where exercise is the the class label for the activity recognition task.

## 4. Experimentation

Our experiments and results are a small subset of the different possibilities that are available. We will discuss a range of available options in the following paragraphs. All of the following topics are intertwined.

### 4.1. Data Segmentation

Due to its considerable length, the complete record of an exercise is unsuitable to show in its entirety to the classifier input. Instead, we divide the data into discrete segments called windows. The body movement data collected using the device’s accelerometer are segmented and the exercise-related data collected using mobile application are used as class label. In each segment, the mode of the class is used for labeling. The research reported in [5,7,10,37,38,39,40,41] experimented with several sliding window lengths with overlap. In this research, we used a window size of 4 s of activity recognition which corresponds to 400 samples. The results are presented without and with overlapping data window. We considered size of window and overlapping on the basis of our experiments. Another reason behind overlapping is that our workout activity is continuous; this overlap ensures that each subsequent window carries some information from the previous window.

Since our task is supervised learning based classification, we need correctly labeled data. Thus, the correct exercise name is tagged with each instance. Furthermore, we also include the exerciser’s subject ID. This information, which is collected separately, has to be correctly appended to each instance.

### 4.2. Classification Algorithm

Our collected dataset can be used with a wide variety of classifier algorithms to train models for the exercise recognition task. Hidden Markov models, support vector machines, naïve Bayes classifiers, and nearest neighbor algorithms are common choices [5,9,39]. The classifier selection depends on many factors, including the input. The input data can be raw signal or extracted higher level features. The sampling rates for data collection and time window of input data are important parameters. The desired comprehensibility of the trained model dictates whether it will be rule-based or not. The processing speed of the trained model from input to output can be a factor. Sometimes, the training time can also be a factor.

Since we have input that is time series data, and raw data are shown in small chunks to the classifier, we use a type of recurrent neural network for our experiments. More specifically, long short-term memory (LSTM) neural networks are used as our model. The main advantage of the LSTM model is its feedback connections. Due to the feedback, an entire sequence of data can be taken under consideration in its calculations. The LSTM consists of three steps: forget part of the previous state, update the memory cell, and output the state. The neurons are arranged in layers and each neuron’s input is multiplied by a weight. The input is scaled layer by layer by means of a transform function. LSTM is an efficient recurrent neural network that integrates the long-term and short-term states of the current processing. Due to its impressive performance, LSTM is extensively used in speech recognition, natural language processing, and image captioning.

The input to the LSTM model are data sequences of fixed length, each containing 400 samples corresponding to 4 s of exercise activity. The LSTM model chooses the class label by using the mode (the value that appears most often) of all exercises which are predicted as present in the sequence.

We use a bidirectional LSTM model that has 128 units; the neurons have relu activation function, and a dropout rate of 0.5. The setup was performed in the TensorFlow with Keras, a commonly used open-source library for machine learning and data mining tasks. Google Colab platform was used for performing the experiments.

### 4.3. Classifier Training

The basis of classifier training is simple enough: identify an exercise that is a member of a given set of exercises. However, there are several options for specifying the exercise samples that would constitute the training set.

The objective of a trained system is to be able to correctly generalize data for a person previously unseen during training. The alternate is to obtain training data from the intended user and then develop a personal model that gives highly accurate results on new data from the same person. In the first case, there is no overhead of individual training before using the system. In the second case, we may potentially have better results. For many applications, only one user is the focus of the system, and in such cases it is better to have personal models if they are resulting in higher accuracy. The training option that we use is to have a mixture of data from several subjects, including the intended user.

The experiment that we are reporting here is a single classifier model, athlete-independent, for all the seven exercises in a workout. Data from all exercises present in a workout group performed by all of the four athletes are used to train and test a single model that is responsible for differentiating between all the exercises in its workout group. Since we have seven exercises/athlete, three sets/week, 6 weeks, and four athletes, this means a total data of 504 sets of exercises for each workout group. There are six workout groups, and thus we have six separate models.

In order to evaluate the performance of classification, the data are split into training and test, based on setting no attribute. As discussed earlier, three sets of each exercise are performed in each workout. The split is based on these three sets of a workout that is performed in a day. The first two sets are reserved for training and the third set for testing. There are a total of 18 sets of an exercise performed by a subject, 12 are made part of the training set and 6 are reserved for the testing set. In other words, we have selected one set of each exercise as test data from each workout performed in a day by an athlete.

(1) $$Accuracy_{}=(\frac{\mathrm{True}\;\mathrm{Positives}+\mathrm{True}\;\mathrm{Negatives}}{\mathrm{Total}\;\mathrm{Evaluations}})$$

(2) $$Precision_{}=(\frac{\mathrm{True}\;\mathrm{Positive}}{\mathrm{True}\;\mathrm{Positive}+\mathrm{False}\;\mathrm{Positive}})$$

(3) $$Recall=(\frac{\mathrm{True}\;\mathrm{Positive}}{\mathrm{True}\;\mathrm{Positive}+\mathrm{False}\;\mathrm{Negative}})$$

(4) $$F_{1}score=2(\frac{\mathrm{Precision}\times \mathrm{Recall}}{\mathrm{Precision}+\mathrm{Recall}})$$

## 5. Results

**Figure 3:**
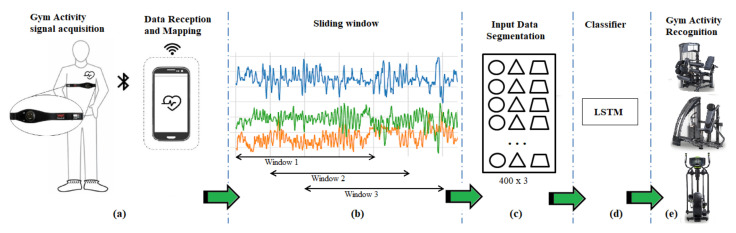
Architecture of the gym physical exercise recognition system, (**a**) data acquisition; (**b**) sliding window; (**c**) input segmentation; (**d**) feeding data to classifier; (**e**) gym activity recognition.

In this section, we present and analyze the results of our experiments. Figure 3 presents the architecture of the proposed activity recognition system following (a) data acquisition from the smart wearable and mobile application; acquired data is labeled with the activity being performed as depicted in Table 3. Step (b) is to create a sliding windows of the raw input signal obtained from the accelerometer. Two datasets are created based on sliding window; the first dataset is non-overlapping and the second is based on overlapping window, as depicted in Figure 3. The next step (c) is input data segmentation, making data ready for the LSTM classifier; in (d) this segment is fed into the LSTM classifier (e) and in the final step, the LSTM outputs the activity being performed for each time window. The LSTM classifier receives this input data segment and outputs the gym activity being performed.

### 5.1. Classifier Testing and Results

The experiments comprise exercise recognition within each of the six muscle groups. A separate model is trained for each muscle group and the training data include data from all the subjects, making the model subject-independent. Our classifiers are trained LSTM models. The commonly used metrics of accuracy (1), precision (2), recall (sensitivity) (3), and F1-score (4) are used to assess the classifiers.

Since the task for each model is to recognize one exercise out of seven, accuracy alone may not be able to give a reliable assessment. Precision allows us to observe how precise our model is, i.e., out of the exercise samples predicted positives, how many are actually positives? Recall, or sensitivity, calculates how many of the actual positives our model has captured among the samples that it has declared as positives. F1 score is another useful measure in the presence of uneven class distribution.

### 5.2. Muscle-Group-Dependent Models

The results for separate models for each muscle group (one model for each muscle group) are presented in Table 4 in term of accuracy and loss. The GAR results are obtained on two datasets; the first is based on non-overlapping window and the second is based on overlapping window. The muscle-group-dependent results for overlapping window are better than the non-overlapping window. The gym workout activity is continuous; this overlap ensures that each subsequent window carries some information from the previous window. This is useful in development of real-time activity recognition applications.

The exercise-wise average of precision, recall, and F-score of separate models for the overlapping dataset are presented in Table 5. Taking into account the fact that only one chest-mounted sensor is used and no effort has been made for optimization of different factors influencing the results, it can be concluded that the experiment is a success with good accuracy, precision, recall, and F-score values. Due to the position of the sensor and the nature of the exercises, the best results are for chest, core body, back, and legs exercises.

**Figure 4:**
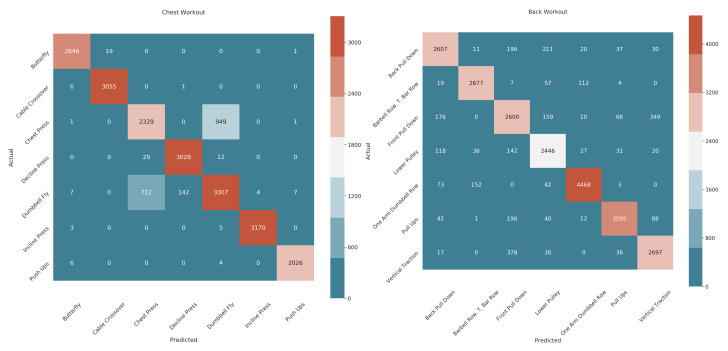
Confusion matrices for activity recognition of chest (on the **left**) and back (on the **right**) workout groups.

The confusion matrices between different exercises of the chest and back muscle group are shown in Figure 4. Both the modules performed very well, with only one exception for the chest workout group. The chest press exercise is confused with dumbbell fly exercise and vice versa. This is due to the fact that both exercises require similar body postures with only a slight change in arm movements. The Zephyr, being a chest-mounted sensor, has difficulty in detecting this subtle difference.

**Figure 5:**
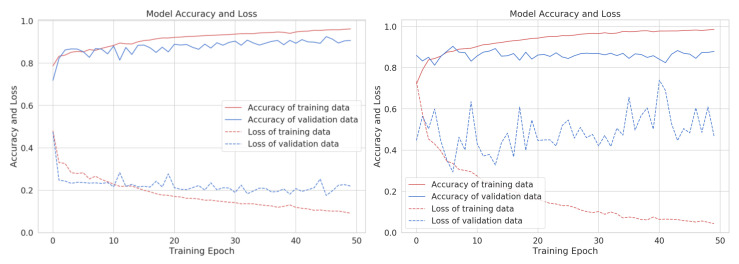
The training and testing evolution for the chest workout (**left**) and back workout (**right**) for each training epoch.

The training and testing evolution for the chest and back workout groups are shown in Figure 5, depicting epoch-wise accuracy and loss of training and validation data for both workout groups. The training and validation accuracy tends to increase and loss tends to decrease with each epoch.

### 5.3. Muscle-Group-Independent Model

**Figure 6:**
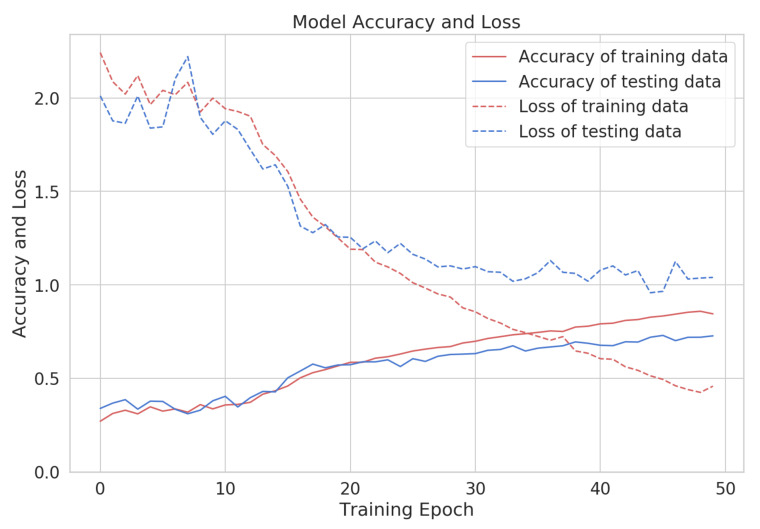
Accuracy and loss for one model for 42 exercises.

**Figure 7:**
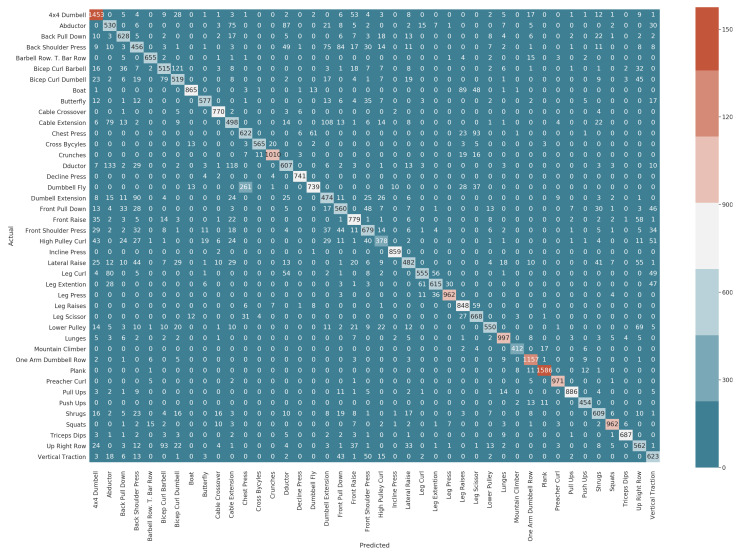
Confusion matrix for one model for 42 exercises.

In the muscle-group-independent model, one model is trained and tested on all the forty-two exercises. The model performed well, with an accuracy of 0.82 and loss 0.61. The results in the form of precision, recall, and F-score for all the exercises are shown in Table 6. The exercises related to chest, back, legs, and core body group performed well. The training and testing evolution is shown in Figure 6. As the model involves forty-two exercises for the gym exercise recognition, we can see in the figure that in the first twenty epochs, the accuracy is low and the loss is high for both training and test data, but with each increasing epoch, the accuracy tends to increase and loss tends to decrease. The confusion matrix for all the exercises is presented in Figure 7.

The muscle-group-dependent models performed well, compared to independent models. One of the reasons is that the muscle-group-dependent models use smaller number of classes, with only seven in each model. The muscle-group-independent model is trained and tested on forty-two classes, which is why its accuracy is low. In practice, it is not possible to perform all the forty exercises in a day, so it is preferred to use muscle-group-dependent models.

## 6. Discussion

Physical activity is widely recognized as one of the important elements of a personal healthy life. To date, with the development of wearable sensing technologies, it is possible to utilize wearable devices and machine learning algorithms to efficiently and accurately monitor physical activity types, intensity, and the associated human pattern for many health applications.

### 6.1. Continuous, Real-Time Processing

For maximum usefulness, an application should be able to handle continuous exercise recognition and also in real time. What more is needed? Imagine that the data are being fed in a continuous stream to the classifier. The most important is detection of intervals between exercises [42]. There might be stillness during transition, and more likely there will be combinations of other activities, such as walking and sitting. It is important for the system to realize when this transition period ends and a regular exercise is detected.

Another aspect is the fast processing of data so that the output keeps up with the input. This can be achieved either by having more powerful computational devices or lighter algorithms for preprocessing and classification, or both. The trend in technology is towards higher computational power packed into smaller, more compact devices. The algorithms that are considered unsuitable today may become feasible tomorrow. Thus, any classifier model that takes an acceptable processing time as defined by the current state of technology can be accommodated, and the frontier keeps on pushing forward with passing time.

### 6.2. Applications of Gym Exercise Recognition

Personal fitness monitoring can make use of exercise recognition, as presented in [5,6,11,16,43]. After developing and testing a system for gym exercise recognition and obtaining good results, the next logical question is, what can be its practical applications? Some major potential applications are discussed below.

#### 6.2.1. Fitness Tracking

An online reporting system can be developed that users can use to trace their fitness-related records and aggregates to gain a greater comprehension of their efforts and the results on a daily, weekly, and monthly spectrum. Such statistics and details can stimulate more fitness-related activity by encouraging users to set targets, make plans, and visualize their improvement. Most importantly, these apps can furnish exercise histories that are not only accurate but are also detailed for end users to help them make statistically sound decisions about their exercise programs. In this way, tedious manual entry of exercise data is avoided. The alternative to manual entry is reliance on memory, which is unreliable and prone to wishful thinking and a distorted perception of what really took place. They can also motivate users by keeping and displaying scores for comparison with their own historical data and other people’s data.

#### 6.2.2. Compliance Monitoring

Gym exercise recognition can also provide a basis for compliance monitoring of patients following exercise programs for rehabilitation by medical personnel, caregivers, and clinicians. The same is true for sport coaches and trainers monitoring trainees. The monitoring personnel will have the ability to monitor by having access to honest remotely generated data, rather than being obliged to be present on the spot.

#### 6.2.3. Trouble Detection and Generation of Alarm

Several alarming situations can happen when a person is training with weights. One of the common events is muscle failure with the trainer stranded under the weights, unable to push them back. If help is not available, it can cause serious injury. An exercise recognition system can possibly detect such failure and generate an alarm at a monitor’s desk (send an alert for help). It would also be able to point out the exercise being performed, thus giving an indication of the subject’s location based on the equipment used for that exercise.

#### 6.2.4. Quality of the Exercises

The quality of an activity’s execution, or how well it was performed can be analyzed, despite the fact that it has the ability to provide relevant information for a wide range of applications. The quality of exercises analysis can help in prevention of injuries and other harmful and wasteful effects of performing exercises in an incorrect way. Automatic assessment of the quality of exercises is a challenging but useful research direction.

#### 6.2.5. Miscellaneous Useful Features

In addition to the applications discussed above, several useful features can be added based on basic gym exercise recognition. For example, we can develop algorithms that can accomplish the following:

- Signal end of resting times between sets and between exercises.
- Automatic monitoring of the intensity of the exercises and estimate of calories burned [39,44,45].
- Disable incoming calls and mute notifications when a trainer is in the midst of an exercise. Such interruptions, even when not attended, can be very distracting. If desired, the user can check his messages and alerts while transitioning between exercises instead of being notified at random during an exercise.
- Play music and change it once an exercise is finished and another one has started.
- Biometrics based on exercises [46].

### 6.3. Limitations of the Study

The data acquisition process involves only four athletes and a single sensor. The chest-mounted sensor results in misclassification of exercises that do not involve chest movement. Additional sensors on other body parts can be used to collect more information. The acquired raw data are fed to the LSTM classifier; by applying feature engineering techniques we can implement other classification algorithms. Although the results presented in this research are good, by overcoming these limitations we can improve gym activity recognition accuracy.

## 7. Conclusions

In this paper, we have presented our experience of collecting and using a dataset for gym activity recognition. Starting from a long process of data collection, spanning over several weeks, we went on to develop models for exercise recognition. We have shared our reflections on the topic and presented some initial results. We have developed several LSTM-based models, and our results are very encouraging. These LSTM models can be used in real-life free weights tracking apps. Furthermore, we can expand the work along, at least, four major axes.

The first axis is the incorporation of more sensors. We have to experiment and understand what different sensors are needed and why. More accelerometers at other places, such as the thigh or wrist, can possibly give better results. Sensors other than accelerometers may also be helpful. However, it is undesirable to have more sensors just for the sake of having more sensors. The selected sensors should work as a team and complement each other for the gym exercise recognition task. With more sensors comes the responsibility of seamlessly synchronizing them together for the classification task.

The second axis is the search for a better classification model. There are scores of models available, each with its advantages and disadvantages. We would like to experiment and analyze several models in the quest for better accuracy.

The third axis is the input to the classifier. Extracted features from the raw data may potentially improve accuracy. It would require extensive experiments and analyses to find the combination of features that are relevant and it would be interesting to discover why they are better than others.

The fourth axis is the development of a continuous, real-time system that not only recognizes the exercises but also automatically counts the repetitions and sets performed for a particular exercise. This would require the system to accurately recognize the transitional period when the subject is not performing exercise and is involved in some other mild activity, such as moving about.

## Figures and Tables

**Table 1:** Weekly workout routine targeting each muscle group.

Sr.	Chest Workout	Arms Workout	Shoulder Workout	Back Workout	Legs Workout	Core Body Workout
1	Butterfly	Bicep Curl Barbell	4 × 4 Dumbbell	Pull Ups	Squats	Leg Raises
2	Cable Crossover	Triceps Dips	Front Raise	Front Pull Down	Leg Press	Crunches
3	Chest Press	Bicep Curl Dumbbell	Back Shoulder Press	Lower Pulley	Leg Extension	Cross Bicycles
4	Decline Press	Dumbbell Extension	Up Right Row	Vertical Traction	Leg Curl	Plank
5	Dumbbell Fly	Preacher Curl	Front Shoulder Press	Back Pull Down	Abductor	Mountain Climber
6	Incline Press	Cable Extension	Lateral Raise	1 Arm Dumbbell Row	Dductor	Leg Scissor
7	Push Ups	High Pulley Curl	Shrugs	Barbell Row. T. Bar Row	Lunges	Boat

**Table 2:** Raw accelerometer data collected using Zephyr device.

Timestamp	Vertical	Lateral	Sagittal
30 December 2019 18:48:02.386	1969	2039	2038
30 December 2019 18:48:02.396	1972	2036	2041
30 December 2019 18:48:02.406	1974	2039	2042
30 December 2019 18:48:02.416	1976	2037	2044
30 December 2019 18:48:02.426	1976	2039	2044

**Table 3:** Labeled body movement and mobile application data.

Timestamp	Vertical	Lateral	Sagittal	User	Wid	Wno	Setno	Exercise
30 December 2019 18:48:02.386	1969	2039	2038	2	2	7	3	Chest Press
30 December 2019 18:48:02.396	1972	2036	2041	2	2	7	3	Chest Press
30 December 2019 18:48:02.406	1974	2039	2042	2	2	7	3	Chest Press
30 December 2019 18:48:02.416	1976	2037	2044	2	2	7	3	Chest Press
30 December 2019 18:48:02.426	1976	2039	2044	2	2	7	3	Chest Press

**Table 4:** Accuracy and loss for separate model of each muscle group.

Muscle Group	Non-Overlapping Dataset		Overlapping Dataset	
	Accuracy	Loss	Accuracy	Loss
Chest	0.81	0.41	0.91	0.26
Arms	0.62	0.97	0.78	1.26
Shoulders	0.57	1.35	0.74	1.40
Back	0.63	0.91	0.88	0.59
Legs	0.75	0.64	0.82	0.81
Core body	0.78	0.56	0.90	0.43

**Table 5:** Exercises average precision, recall, and F-score based on muscle group.

Muscle Group	Exercises Average		
	Precision	Recall	Fscore
Chest	0.924	0.924	0.924
Arms	0.767	0.767	0.765
Shoulders	0.734	0.730	0.730
Back	0.872	0.873	0.872
Legs	0.806	0.807	0.806
Core body	0.895	0.883	0.888

**Table 6:** Precision, recall, and F-score for the model covering all exercises.

Exercise Name	Precision	Recall	Fscore	Exercise Name	Precision	Recall	Fscore
4 × 4 Dumbell	0.824	0.890	0.856	High Pulley Curl	0.690	0.559	0.618
Abductor	0.569	0.658	0.610	Incline Press	0.982	0.997	0.989
Back Pull Down	0.775	0.820	0.797	Lateral Raise	0.724	0.578	0.643
Back Shoulder Press	0.540	0.566	0.553	Leg Curl	0.851	0.678	0.755
Barbell Row. T. Bar Row	0.955	0.947	0.951	Leg Extention	0.853	0.775	0.812
Bicep Curl Barbell	0.679	0.646	0.662	Leg Press	0.957	0.947	0.952
Bicep Curl Dumbell	0.659	0.679	0.669	Leg Raises	0.810	0.912	0.858
Boat	0.958	0.846	0.898	Leg Scissor	0.718	0.895	0.797
Butterfly	0.920	0.828	0.872	Lower Pulley	0.872	0.709	0.782
Cable Crossover	0.923	0.971	0.947	Lunges	0.918	0.938	0.928
Cable Extension	0.570	0.623	0.595	Mountain Climber	0.965	0.934	0.949
Chest Press	0.665	0.771	0.714	One Arm Dumbbell Row	0.918	0.967	0.942
Cross Bycyles	0.972	0.920	0.946	Plank	0.980	0.980	0.980
Crunches	0.969	0.947	0.958	Preacher Curl	0.977	0.986	0.981
Dductor	0.681	0.640	0.660	Pull Ups	0.976	0.938	0.956
Decline Press	0.976	0.987	0.981	Push Ups	0.925	0.946	0.935
Dumbbell Fly	0.895	0.679	0.772	Shrugs	0.765	0.768	0.767
Dumbell Extension	0.574	0.646	0.608	Squats	0.958	0.934	0.946
Front Pull Down	0.667	0.678	0.673	Triceps Dips	0.977	0.945	0.961
Front Raise	0.769	0.823	0.795	Up Right Row	0.642	0.673	0.657
Front Shoulder Press	0.704	0.707	0.705	Vertical Traction	0.668	0.795	0.726

## Data Availability

The raw data supporting the conclusions of this article will be made available by the corresponding author upon reasonable request.

## Abbreviations

The following abbreviations are used in this manuscript:

IoT	Internet of Things
LSTM	Long short-term memory
GAR	Gym activity recognition
HAR	Human activity recognition
IMU	Inertial measurement unit
FEMO	Free weight exercise monitoring system
BH3	Zephyr BioHarness 3
